# Genotype and chemotype insights of high-THC medicinal *Cannabis sativa* L.: the role of SSR markers in the identification of cultivars

**DOI:** 10.1186/s42238-025-00357-w

**Published:** 2025-11-22

**Authors:** Ana Patrícia Gomes, Sara Vicente, Joana Rosa, Iva Vinhas, António Marques da Costa, Michael Sassano, Luis Monteiro Rodrigues, Patrícia Rijo, Helena Trindade, Maria do Céu Costa

**Affiliations:** 1 CBIOS - Universidade Lusófona’s Research Center for Biosciences & Health Technologies, Campo Grande 376, Lisboa, 1749-024 Portugal; 2 SOMAÍ Pharmaceuticals, R. 13 de Maio 52, Carregado, 2580-507 Portugal; 3 NICiTeS, IPLUSO, ERISA-Escola Superior de Saúde Ribeiro Sanches, Lisboa, Portugal; 4 cE3c - Centre for Ecology, Evolution and Environmental Changes & CHANGE - Global Change and Sustainability Institute, Faculdade de Ciências, Universidade de Lisboa, Campo Grande, Lisboa, 1749-016 Portugal

**Keywords:** *Cannabis sativa* L., SSR marker, Cannabinoids, Flavonoids, Phenolics, Cultivar fingerprinting, Therapeutic applications

## Abstract

**Background:**

Standardized methods for distinguishing high-THC *Cannabis sativa* L. genotype versus chemotype of cultivars remain limited, posing challenges for quality control and regulatory compliance in the medicinal cannabis industry. This study addresses the integration of genetic and chemical profiling for the standardization and quality control of medicinal High-THC *Cannabis sativa* L. clonal cultivars, focusing on the potential use of SSR markers combined with high-performance liquid chromatography (HPLC) for cultivar characterization.

**Methods:**

DNA was extracted from *C. sativa* L. leaf using a modified Mini-CTAB protocol, followed by PCR amplification based on 12 SSR primers, and capillary electrophoresis for genetic analysis. HPLC-PDA was used to quantify cannabinoid content, while non-cannabinoid components, including phenolics, flavonoids, chlorophyll, and waxes, were investigated using specific methods. Using eight individual specimens of the plants, the study evaluated three distinct cultivars (identified as A, B, and C), representing key genetic variations.

**Results:**

Genetic analysis revealed that the eight SSR markers amplified allele sizes ranging from 147 to 326 bp, depending on the locus. ANUCS303, ANNUCS304, and C11CANN1 amplified respectively two, three, and three alleles and were valuable tools for cultivar fingerprinting. Further validation based on a broader range of cultivars and primers is needed to enhance the reliability of SSR-based cultivar identification. Chemical analysis showed similar cannabinoid profiles across cultivars, with a high THC [82–84% (w/w)] and a low CBD [0.5–0.8% (w/w)]. Non-cannabinoids, including waxes and chlorophylls, were quantified and efficiently removed during process purification. The extraction and purification processes significantly increased ∆^9^-THC content, with total cannabinoid content reaching 97% in purified extracts while preserving cannabinoid profiles, with minor differences across the cultivars’ chemical fingerprints.

**Conclusions:**

The study highlights the importance of combining genetic and chemical profiling for standardizing *C. sativa* L. cultivars. SSR markers can effectively aid in cultivar identification and quality control, and the combined “identity fingerprint” is important to ensure therapeutic equivalence across batches of clonal lines. These efforts should be designed to enhance the safety, reliability, and efficacy of vegetatively propagated cannabis-based therapeutics.

**Supplementary Information:**

The online version contains supplementary material available at 10.1186/s42238-025-00357-w.

## Introduction

*Cannabis sativa* L. is a highly versatile plant with significant applications across medicinal, industrial, and recreational contexts, having been known and used for thousands of years (Henry et al. 2020). Industrially, *C. sativa* L. has been cultivated for its fiber and seeds, while its psychoactive effects have been sought for recreational and religious purposes. In medicinal contexts, its unique phytochemical composition, particularly the cannabinoids THC (Δ^9^-tetrahydrocannabinol) and CBD (cannabidiol), has shown therapeutic potential for various conditions (Booth and Bohlmann 2019; Li 1973; Salami et al. 2020).

These cannabinoids may interact with cannabinoid receptors of the endocannabinoid system, due to their similar structure to endocannabinoids, therefore contributing positively to the quality of life and well-being of the population where conventional treatments have fallen short, especially in nervous system disorders, underscoring a critical medical opportunity (Gonçalves et al. 2019). More importantly, it was proven that these cannabinoids alone were not as efficient as the whole plant chemical drug preparation (Berman et al. 2018; Blasco-Benito et al. 2018; Gallily et al. 2015; Johnson et al. 2010; Nallathambi et al. 2018). The rich chemical biodiversity within.

*Cannabis sativa* includes not only cannabinoids but also terpenes, polyphenols, flavonoids, chlorophylls, and fatty acids, among other bioactive compounds (Committee on Herbal Medicinal Products (HMPC), 2021; Jin et al. 2020; Pattnaik et al. 2022) totaling over 200 phytocannabinoids identified to date (Govindarajan et al. 2023; Hussain et al. 2021; Micalizzi et al. 2021). These compounds are referred to as contributing to what is known as the “entourage effect”, where their synergistic interactions among these compounds could enhance therapeutic outcomes, therefore presenting exciting possibilities for maximizing treatment efficacy (André et al. 2024). However, the complexity of these interactions is neither proven nor clearly understood, highlighting the importance of accurately understanding and identifying cannabis chemotypes and genotypes, particularly to ensure consistent quality and efficacy in medicinal applications.

Traditional labels, such as *‘indica’* and *‘sativa*’, often fail to reflect the plant’s chemical or genetic composition, underscoring the need for precise characterization of *C. sativa*’s strains (Adams et al. 2021; Booth and Bohlmann 2019). Besides this, and despite its therapeutic promise, *C. sativa* also faces a significant challenge in the pharmaceutical industry, such as variations in chemical profiles between production cycles resulting from its high heterozygosity (Adams et al. 2021), and sensitivity to environmental factors (Gorelick et al., 2017). The secondary metabolite profiles differ considerably between plant organs (Bernstein et al. 2019) and are strongly influenced by abiotic stresses such as salinity and heat (Baas et al., 2023; Rodriguez-Morrison et al. 2021), plant structural traits (Danziger et al., 2021), mineral nutrition (Song et al. 2023) and planting density (Danziger et al., 2022).

Such inconsistencies pose issues for medical applications, where reproducible chemotypes are essential to ensure therapeutic efficacy, reliability, and patient safety (Fulvio et al. 2023). To overcome this challenge, many commercial cultivar suppliers have developed proprietary clones, which are genetically uniform plants derived from a single parent through cloning. These clones preserve a consistent genotype and thus, supposedly a more stable chemotype (Reimann-Philipp et al. 2020). The pharmaceutical industry minimizes the impact of environmental conditions with the “indoor” cultivations of these single-strain cultivars, where conditions such as light intensity and cycles, environmental temperature, CO_2_ concentrations, and nutrients are controlled and standardized for each strain’s/cultivar´s growing conditions. Therefore, as the cannabis market expands, it is increasingly important to accurately differentiate between the genetic profiles of these cultivar-specific clones, ensuring product authenticity and compliance with pharmaceutical standards.

Molecular markers are essential tools for cannabis characterization, enabling the analysis of genetic diversity, cultivar identification, and breeding strategies (Schwabe & Mcglaughlin 2019). Initially, markers like Random Amplified Polymorphic DNA (RAPD) and Amplified Fragment Length Polymorphism (AFLP) were widely used, but the RAPD’s lower reproducibility and specificity and the labor involved in AFLP have made them less favorable compared to more advanced markers like Simple Sequence Repeats (SSRs) and Single Nucleotide Polymorphisms (SNPs) (Abdelhamid et al. 2024; Miller Coyle et al. 2003; Vyhnánek et al. 2020). Microsatellites or SSRs particularly stand out for providing detailed genetic insights per locus without the need for high-throughput technologies or extensive computational resources as required by SNPs (Alghanim and Almirall 2003; Borin et al. 2021; Gilmore and Peakall 2003).

Microsatellites have become the gold standard in cannabis genetic studies, widely used to create genetic fingerprints, assess genetic similarity, and ensure clonal fidelity, critical for maintaining stable chemotypes in commercial cultivars (Rawls et al. 2015). Their applications span evaluating genetic variability, managing germplasm, and ensuring regulatory compliance (Alghanim and Almirall 2003; Dufresnes et al. 2017; Gilmore et al. 2003; Hsieh et al. 2003). As cannabis continues to expand in medical and commercial sectors, accurately tracing genetic profiles is essential for ensuring product authenticity and quality control.

To tackle these known challenges, this research underscores the significance of scrutinizing the chemical composition of strains in cultivation practices. Specifically, applying SSR markers to assess and distinguish between different cannabis strains offered by diverse cultivars will enable the development of profiles for each cultivar’s strain, streamlining the process of recognizing and confirming distinct genetic profiles. These unique genetic markers serve not only to verify the legitimacy of varieties utilized for medical purposes but also aid in maintaining the necessary quality control protocols for consistency. This genotype-chemotype relationship is especially valuable for the pharmaceutical industry, as it enables the production of true-to-type plants with reproducible phytochemical profiles, essential for regulatory compliance and therapeutic consistency.

In this study, firstly, we aim to analyze the genetic variability among *C. sativa* cultivars of SOMAI using SSR markers. We hypothesized that the SSR markers used could reliably differentiate cultivars, thereby establishing distinct genetic fingerprints for each cultivar. Secondly, we aim to determine the cannabinoid and non-cannabinoid content of these cultivars, both before and after purification, providing insights into the chemical biodiversity inherent in *C. sativa* different cultivars. This work seeks to provide advances in the standardization, improved traceability, and quality control of medicinal cannabis, ultimately enhancing its safety and effectiveness in therapeutic applications. One main objective was to address the regulatory compliance in the medicinal cannabis industry. This exploratory study needs further molecular studies to address the genotype-chemotype associations, examining specific genetic profiles and correlating with the production of cannabinoids and other bioactive compounds.

## Materials and methods

### Plant material

High-THC *Cannabis sativa* L. plant samples were supplied by SOMAI Pharmaceuticals (Carregado, Portugal), consisting of *Cannabis* flos (cannabis flower), dried, whole or fragmented, fully developed inflorescences from female *C. sativa* L. The samples, all compliant with the DAB monograph for this herbal substance, were obtained from eight batches of plants at the flowering stage (brown and green flowers) belonging to three distinct cultivars: cultivar A (1–3), cultivar B (4–5), and cultivar C (6–8). Each cultivar originated from a different supplier (grower), and each cultivar was propagated through stem cuttings (vegetative propagation), so plants from one cultivar were true clones, with genetic uniformity.

The plant material followed the standard harvesting process, in which plant maturity is determined by visual inspection of flower trichomes and their color. Harvesting was performed manually by trained personnel, either by cutting individual flowers or entire branches. All samples were collected at an equivalent maturation stage.

The inflorescences were trimmed (mechanical + manual trimming) immediately after harvest and submitted to drying using trays, in the absence of light, with ventilation at low temperature (max. 20 °C), controlled Relative Humidity (HR: 50%), and for up to 264 h.

The leaves used for DNA testing corresponded to the remaining “sugar leaves” (small leaves borne within the inflorescence) that were not fully removed during trimming of the inflorescences. These leaves, according to the Ph. Eur. Pharmacopoeia monograph, are considered as part of “foreign matters” with a general limit of less than 2%.

All the cannabis plants from which the inflorescences were obtained were grown in greenhouses with temperature-controlled conditions and hydroponic solutions with nutrient composition controlled at each step of the process (Table 1). Suppliers (Portuguese qualified growers) comply with GACP (WHO/EMA Good Agricultural and Collection Practices) accreditation and have been previously audited by the pharmaceutical company producer, SOMAÍ, for qualification purposes.

**Table 1:** *Cannabis sativa* cultivation conditions in three growers, including the nutrient concentrations (%), temperature (ºC), pH and relative humidity (% RH), and drying conditions

Critical Steps	Stage of the Herbal Substance Manufacturing Process		
	Vegetative	Flowering	Drying
**Light/Darkness cycle**	≈ 18–24 h of light	≈ 12 h of light	Lighting is to be minimized during drying
**Temperature (ºC) and Relative Humidity (% RH)**	25–30 °C (day) 20–24 °C (night) 60–75% RH	24–30 °C (light) 18–24 °C (darkness) 40–60% RH	19 ± 2 °C ≤ 50%±10% RH
Nutrient* levels **(EC) and pH**	*Macronutrients*: N, NH_4_, NO_3_, P_2_O_5_, K_2_O, CaO, MgO, SO_4_ *Micronutrients*: Fe, Mn, Zn, Cu, Mo, B *pH in the substrate*: 5.5–6.5	Macronutrients** N, NH_4_, NO_3_, P_2_O_5_, K_2_O, CaO, MgO, SO_4_ Micronutrients** Fe, Mn, Zn, Cu, Mo, B *pH in the substrate*: 5.5–6.5	Not applicable

*Nutrient run-off is tested on each watering cycle for electrical conductivity. Examples of nutrients used: monoammonium phosphate 12–61−0 (65% P_2_O_5_, 27% P, N total 12%, 12% N-NH_4_) or monoammonium phosphate N 12% P_2_O_5_ 61.4%; calcium nitrate 15.5-0-0 + 26.5 CaO (15.5% N, 19% Ca, N-NH_3_ 14.4%, N-NH_4_ 1.1%, CaO 26.5%) or calcium nitrate 17-0-0 (33; magnesium Nitrate [Mg (NO_3_)_2_.6H_2_O] or magnesium nitrate 10.5% N (Nitric)/15.6% MgO; potassium Sulfate 0-0-52-15 (S) or potassium sulfate 52% K_2_O/45% SO_3_

**Different stages of nutrient concentration are verified during flowering (immature, flowering, and later stages), followed by flushing before harvesting

Environmental and nutritional controls are optimized and standardized by each grower and ensured by a technical agreement signed. All the strains used are THC-dominant (50% *C. sativa*/50% *C. indica*) in compliance with European Monograph 3028 (Council of Europe, 2024). The reproducibility between batches of the same grower supports the assumption of well-controlled growing conditions. Cannabis flowers were brown-green, clustered flowers of 0,5–5 cm, specifications which complied with all requirements of the German DAB Monograph for Cannabis Flos (German Pharmacopoeia 2022).

### DNA isolation and quantification

DNA was extracted from leaf samples via organic solvents using a modified Mini-CTAB protocol based on (Doyle and Doyle 1987), modified by (Weising et al. 1994) and adapted as follows: Approximately 35–60 mg of dried leaves were grounded to a fine powder using a ball mill, and each sample was mixed with preheated CTAB (cetyltrimethylammonium bromide) buffer (2%, 65 °C) containing PVP-40 (Polyvinylpyrrolidone) and *β*-mercaptoethanol to reduce polyphenol oxidation. Chloroform: isoamyl alcohol (24:1) was added to aid in phase separation, and samples were incubated at 65 °C for 30 min and centrifuged. The aqueous phase was collected, and DNA was precipitated with cold ethanol (−20 °C). The pellet was washed with ammonium acetate in 76% ethanol, air-dried, and resuspended in TE buffer. DNA concentration was assessed using a Nanodrop spectrophotometer (ThermoScientific) based on A260, while purity was evaluated through A260/280, and A260/230 absorbance ratios. Stock DNA samples were stored at −20^ο^C, and the DNA working samples were diluted to 10 ng/µL in TE buffer and stored at 4^ο^C.

### PCR reaction and optimization

PCR reactions were carried out in a total volume of 20.0 µL, with 30 ng of genomic DNA. The NZYTech green master mix was used to prepare the reaction mix, with minor modifications in the MgCl₂ concentration, which we initially varied from 2.5 mM (as provided in the master mix) to 4 mM. PCR amplification was carried out with the 12 microsatellite primers listed in Sect. 2.1.1, and optimal conditions were found to be 2.5 mM of MgCl_2_ for primers B01CANN1 and B05CANN1, and 3 mM for primers C11CANN1, ANUCS301, ANUCS303, ANUCS304, ANNUCS201, ANUCA501, and H09CANN2 (Ioannidis et al. 2022).

PCR amplification was carried out in a BioRad T100 thermocycler with the conditions optimized for each primer set. Each PCR setup featured a negative control containing all reaction components but without a DNA template. All reactions started with an initial denaturation step at 94 °C for 10 min and a final extension at 72 °C for 15 min. PCR conditions for each set of primers can be found in Table 2. A touchdown PCR was performed to decrease non-specific amplification, as described in conditions 1 and 2.

**Table 2:** PCR conditions for *C. sativa* SSR analysis. Each column represents a distinct PCR protocol (Conditions 1, 2, and 3) tailored to specific primer sets. All protocols included an initial denaturation at 94 °C for 10 min and a final extension at 72 °C for 15 min

PCR Conditions	Condition 1	Condition 2	Condition 3
Initial Denaturation	94 °C, 10 min	94 °C, 10 min	94 °C, 10 min
First Conditions	10 cycles	10 cycles	35 cycles
Denaturation	94 °C, 30 s	94 °C, 30 s	94 °C, 30 s
Anneling	60 °C, 60 s	60 °C, 30 s	60 °C, 60 s
Extension	72 °C, 60 s	72 °C, 60 s	72 °C, 60 s
Touchdown	30 cycles	30 cycles	Not applied
Denaturation	94 °C, 30 s	94 °C, 30 s	-
Anneling	55 °C, 60 s	55 °C, 30 s	-
Extension	72 °C, 60 s	72 °C, 60 s	-
Final extension	72 °C, 15 min	72 °C, 15 min	72 °C, 15 min
Primers	C11 CANN ANUCS 301 ANUCS 303 ANUCS 304	B01 CANN1 B05 CANN1	ANNUCS 201 ANNUCS 501 H09 CANN2

### Agarose gel electrophoresis and capillary electrophoresis

Successful PCR amplification was confirmed by running an agarose gel with Xpert Green as a DNA stain, allowing visualization of the amplified products. Primers yielding visible and reproducible amplification of expected sizes were selected for further analysis through capillary gel electrophoresis. Primers labeled with fluorescent dyes (Hex or 6-FAM) were used for PCR amplification. Capillary gel electrophoresis was performed by STABVIDA (Costa da Caparica, Portugal), and fragments were analyzed using the Peak Scanner software in the Thermo Fisher Connect platform. All samples were analyzed in duplicate, with each PCR reaction set including one negative and one positive control.

### Sample Preparation for HPLC quantification

Cannabis flower samples were prepared by weighing 500 mg of dried, ground material into a 50 mL falcon tube (protected from light), followed by the addition of 20 mL of ethanol (96%, Carlo Erba). The mixture was mixed at 300 rpm for 15 min and centrifuged at 3500 rpm for 5 min (VWR Mega Star 600). The clear supernatant was transferred to a volumetric flask, and the process was repeated twice as much with 12.5 mL of ethanol (96%) each time. The final volume was adjusted to 50 mL with ethanol, then filtered the solution through a 0.45 μm membrane, and finally took a 1 mL aliquot of the extract and diluted it to 10 mL with ethanol in a volumetric flask. Two independent solutions were prepared for the ground flower.

For the analysis of raw and purified extracts, 20 mg of product was weighed into a 100 mL volumetric flask and diluted to volume with ethanol (96%). The solution was vortexed (VWR I444-1372) until completely dissolved and filtered through a 0.22 μm membrane filter. Two independent solutions of each raw and purified extract were prepared.

### Cannabinoids quantification

Cannabinoid analysis was conducted using High-Performance Liquid Chromatography (HPLC), equipped with a Photodiode Array Detector (PDA). The system included a Waters 2998 PDA Detector, a Quaternary Pump, a Quaternary Solvent Manager R, and an Autosampler (Waters Sample Manager FTN-R), all managed with Waters Empower software (version 3.7.0). Chromatography was performed using a pre-column (5 mm × 3 mm, octadecylsilylized silica gel, 2.7 μm) and an analytical column (150 mm × 3 mm, octadecylsilylized silica gel, 2.7 μm) at a flow rate of 1.0 mL min⁻¹.

The mobile phase consisted of solvent A (aqueous phosphoric acid, 8.64 g/L) and solvent B (acetonitrile), with an initial gradient elution of 64% B. Over 16 min, the gradient gradually increased to 82% B. The proportion of solvent B was then reduced to 64% for 1 min and held constant for 3 min to allow for re-equilibration. The column temperature was maintained at 40 °C, and the autosampler was set to 5 °C. The injection volume was 10 µL for both samples and standards. UV detection was performed at 225 nm for non-acid cannabinoids and 306 nm for acid cannabinoids.

The reagents used to determine the content of cannabinoids by HPLC-PDA included ultrapure water (Milli-Q) supplied by Milli-Q IQ 7000 Merck system, ethanol 96% (V3A033063B), acetonitrile (HPLC grade, P3C653153C), and methanol (HPLC grade, V2N046222N), all provided by Carlo Erba. Phosphoric acid (L2440) was sourced from Honeywell. The reference standards used in the analytical methods were Cannabidivarin (CBDV, 2-H444030ME), Cannabidiolic acid (CBDA, 2-H495778AL), Cannabigerolic acid (CBGA, 2-H444251AL), Cannabigerol (CBG, 2-H497325ME), Tetrahydrocannabivarinic acid (THCVA, 2-H497522AL), Cannabichromene (CBC, 2-H542472AL), Cannabicyclolic acid (CBLA, 2-H441929AL), and Cannabichromenic acid (CBCA, 2-H497522AL), all provided by DR EHRENSTORFER. Additionally, Cannabinol (CBN, A0177171), Δ⁹-Tetrahydrocannabinol (Δ⁹-THC, A0180155), and Δ⁸-Tetrahydrocannabinol.

(Δ⁸-THC, A0180815) were supplied by RESTEK.

### Calibration curve

Calibration curves were generated using standard solutions prepared in methanol. Stock solutions included Δ⁹-THC and CBD (500 µg/mL, Stock Solution A), Δ⁹-THCA and CBDA.

(500 µg/mL, Stock Solution B), and CBN (20 µg/mL, Stock Solution C). For flower samples, the system standard solution (SSS) was prepared by combining stock solutions A, B, and C to achieve final concentrations of Δ⁹-THC and CBD at 10 µg/mL, Δ⁹-THCA and CBDA at 50 µg/mL, and CBN at 1 µg/mL. For extracts, calibration standards included Δ⁹-THC, CBD, and CBN at 200 µg/mL, 200 µg/mL, and 2 µg/mL, respectively, and Δ⁹-THCA and CBDA at 20 µg/mL. Cross-verification standard solutions were independently prepared to validate calibration robustness. Calibration curves demonstrated linearity (R² ≥ 0.99), and the relative standard deviation (RSD) of response factors was ≤ 5%.

### Non-cannabinoids content

Total phenolic content was assessed using the Folin-Ciocalteu method (Cicco et al. 2009; Kupina et al. 2018; Waterhouse 2003). The calibration curve was determined from five standard solutions prepared using gallic acid (Sigma-Aldrich) with concentrations between 50 and.

500 mg/L. In brief, 100 µL of Folin-Ciocalteu reagent (Sigma-Aldrich) and 1,58 mL of water were added to 20 µL of 100 mg/mL extract in methanol (MeOH, Carlo Erba). The mixture was incubated for 7 min, and 300 µL of a 20% sodium carbonate (Na_2_CO_3_, Sigma-Aldrich) was added. The absorbance was measured at 750 nm after incubating at 40 °C for 30 min.

Total flavonoid content (Navarro et al. 2015; Shraim et al. 2021) was determined with a calibration curve established by using five standard solutions with concentrations ranging from 5 to 250 mg/L of quercetin (Sigma-Aldrich). In short, 37.5 µL of 5% sodium nitrite (NaNO₂, Merck) was added to 125 µL of the extract at 100 mg/mL. After incubating for 6 min at room temperature, 75 µL of 10% aluminum chloride (AlCl₃, Sigma-Aldrich) was added and allowed to incubate for 5 min more. Finally, 250 µL of sodium hydroxide (NaOH, 1 M, Merck) was added, and distilled water was used to bring the total volume to 1.25 mL. Absorbance was measured at 510 nm.

Total chlorophyll content (Arnon 1949; Tiago et al. 2022) was determined by diluting.

0.1 g of extract in 10 mL of 96% ethanol and centrifuged at 6000 rpm for 15 min. The absorbance of the supernatants was measured at 663 nm and 645 nm, and the contents of chlorophyll a (CHLa), chlorophyll b (CHLb), and total chlorophyll (Total CHL) were determined by formulas (1–3).

1 $$\:{\text{C}\text{H}\text{L}}_{a}=\:(12.25\times\:{Abs}_{663nm\:})\:-\:(2.79\times\:{Abs}_{645nm\:})\:\:\:\:\:\:\:\:\:\:\:\:\:\:\:\:\:\:\:\:\:\:\:\:\:\:\:\:\:\:\:\:\:$$

2 $$\:{\text{C}\text{H}\text{L}}_{b}=\:(21.50\times\:{Abs}_{645nm\:})\:-\:(5.10\times\:{Abs}_{663nm\:})\:\:\:\:\:\:\:\:\:\:\:\:\:\:\:\:\:\:\:\:\:\:\:\:\:\:\:\:\:\:\:\:\:$$

3 $$\:{\text{C}\text{H}\text{L}}_{total}=\:{\text{C}\text{H}\text{L}}_{a}+\:{\text{C}\text{H}\text{L}}_{b}\:\:\:\:\:\:\:\:\:\:\:\:\:\:\:\:\:\:\:\:\:\:\:\:\:\:\:\:\:\:\:\:\:\:\:\:\:\:\:\:\:\:\:\:\:\:\:\:\:\:\:\:\:\:\:\:\:\:\:$$

Wax quantification (Tiago et al. 2022) was determined by precipitating the waxes, namely, 100 mg of the sample was dissolved in 10 mL of 96% ethanol and incubated for 24 h at.

−20 °C. The mixture was centrifuged at 6000 rpm for 15 min, and the resulting pellet was left to dry overnight. The solvent from the supernatant was subsequently removed using a rotary evaporator. The total wax content is calculated as follows (4):

4 $$\:{\text{W}\text{a}\text{x}\text{e}\text{s}}_{total}\:\left(\%\right)=\:\frac{Extracted\:waxes\:\left(g\right)}{Feed\:mass\:\left(g\right)}\:\:\times\:100\:\:\:\:\:\:\:\:\:\:\:\:\:\:\:\:\:\:\:\:\:\:\:\:\:\:\:\:\:\:$$

All experiments were performed in triplicate and presented in percentage (%) of each non-cannabinoid content.

### Statistical analysis

Statistical analyses were performed in R (R Core Team, 2016). Group comparisons were conducted using the Kruskal–Wallis test, followed by Dunn’s post-hoc test with *p*-values adjusted using the Benjamini–Hochberg method, and *p*-value < 0.05 considered statistically significant. Box plots are provided in the supplementary material (Figures S3 and S4) to illustrate the results of statistically significant differences.

## Results and discussion

### Genetic analysis with SSR markers

DNA extractions yielded high-quality DNA based on the assessment of the A260/A280 ratio (between 1.8 and 2.0), and similar quantities among extractions. From the initial 12 primer set, nine primers were selected for further analysis via capillary gel electrophoresis. The PCR conditions for three primers (ANUCS 202, ANUCS 302, and ANUCS 305) could not be optimized, and several fragments were obtained. The selected nine primers yielded fragments ranging from 150 to 300 bp and produced clear and reproducible bands.

**Fig. 1:**
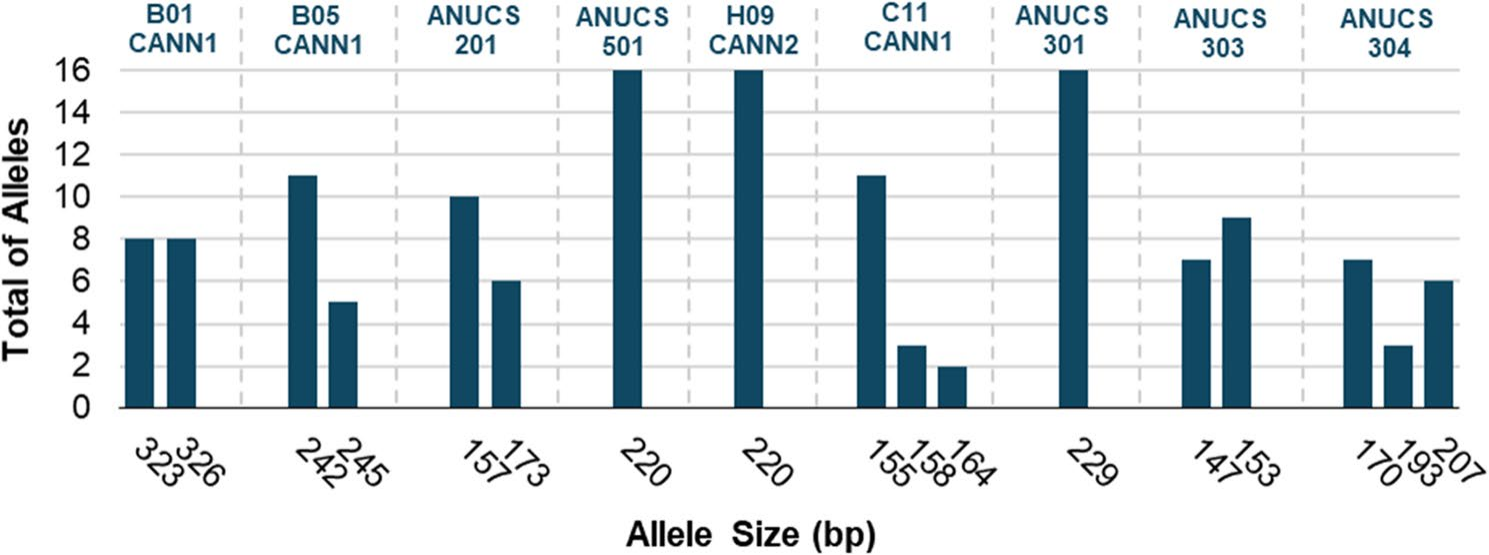
Summarized analysis of the distribution of allele sizes in base pairs (bp) detected across loci using SSR markers in capillary electrophoresis

Capillary gel electrophoresis following PCR amplification using fluorescently labeled primers provided the correct allele size, revealing that the primers amplified between 1 and 3 different alleles per locus, considering the plants under study. Allele sizes ranged from 147 to 326 bp, depending on the SSR locus Fig. 1.

In general, allele frequency was variable in the sampling under study, namely, primers ANUCS301, ANUCS501, and H09CANN2 amplified only one allele, primers C11CANN1 and ANUCS304 revealed three alleles in the plants under study, while the remaining primers amplified two alleles.

Notably, four primers, B01CANN1, H09CANN2, ANUCS 301, and ANUCS 501, showed no genetic differences between the plants from the different cultivars, reflecting loci that are highly conserved and less informative for distinguishing cultivars. Additionally, all samples were found to be homozygous using primers ANNUCS 301, ANNUCS 501, and H09CANN2, but conversely were all found to be heterozygous using primer B01CANN1. This heterozygosity did not provide enough genetic distinction to separate the cultivars, as both allele sizes were shared by all plants, suggesting that although the alleles were polymorphic, the variation was not sufficient to differentiate cultivars in this context. Further analysis was made to determine the ability of specific primers to distinguish between plants from different cultivars (Figure S1 of the supplementary materials).

Notwithstanding, primers C11CANN1, ANUCS201, ANUCS303, ANUCS304, and B05CANN1 exhibited greater genetic diversity amongst the cultivars, while maintaining consistent amplification patterns within accessions from the same cultivar. For example, primer C11CANN1 revealed distinct allelic patterns for each cultivar, with alleles 155 and 158 characterizing cultivar A (Plants 1, 2, and 3), alleles 155 and 164 were exclusive to cultivar B (Plants 4 and 5), and allele 164 was unique in cultivar C (Plants 6, 8, and 9). Similarly, ANUCS303 exhibited cultivar-specific profiles, with homozygosity for allele 147 in cultivar B and for allele 153 in cultivar C, and heterozygosity (147 and 153) in cultivar A. For cultivar A, ANUCS304 amplified alleles 170 and 193, while in cultivars B and C, only the homozygous alleles 170 and 207 were amplified, respectively.

Overall, the molecular analysis confirmed that plants from the same cultivar shared identical alleles for all primers tested, proving their genetic similarity. Despite some loci displaying conserved alleles, certain primers revealed sufficient genetic variability to distinguish between cultivars. Among the primers evaluated, ANUCS303, ANUCS304, and C11CANN1 demonstrated the greatest ability to differentiate cultivars, with clear allelic patterns that distinguished all three groups. These primers exhibited both heterozygosity and cultivar-specific allele profiles, making them the most effective in this study for preliminary cultivar fingerprinting. It is important to note that the results are based on a small sample size of eight plants from three cultivars. Therefore, these findings should be considered preliminary, and additional testing with a larger sample size is necessary to validate the observed patterns and further refine the ability of these primers to discriminate between more cultivars.

Furthermore, allele sizes observed in this study fell within the size ranges reported in the literature (Alghanim and Almirall 2003; Gilmore et al. 2003; Gilmore and Peakall 2003; Houston et al. 2017; Howard et al. 2008; Ioannidis et al. 2022; Köhnemann et al. 2012), with some minor deviations (Table 3).

**Table 3:** Allele size range (bp) obtained and expected for the eight optimized microsatellite loci

Primers	Allele size range obtained (bp)	Expected Allele size range (bp)
C11CANN1	155–164	152–167^A^ 150–175^B^ 151–176^C^ 138–180^D^
ANUCS501	220	90 ^A^ 88–98 ^D^ 80–95 ^F^ 72–105 ^G^
B05CANN1	242–245	244–256 ^A^ 235–244 ^B^ 236–245 ^D^ 217–243 ^G^
B01CANN1	323–326	324–327 ^A^ 323–339 ^B^ 314–349 ^C^ 317–329 ^D^ 335–460 ^G^
ANUCS303	147–153	147 ^A^ 145–151 ^D^ 142–157 ^E^ 141–156 ^F^
H09CANN2	220	205–219 ^A^ 204–224 ^B^ 204–221 ^C^
ANUCS304	170–207	146–209 ^A^ 171–207 ^D^ 167–230 ^E^
ANUCS201	157–173	182–204 ^A^ 155–227 ^E^ 161–223 ^F^

**(A)** (Ioannidis et al. 2022), (B) (Alghanim and Almirall 2003), **(C)** (Köhnemann et al. 2012), (D) (Köhnemann et al. 2012), (E) (Gilmore et al. 2003), (F) (Gilmore and Peakall 2003), **(G)** (Xia et al. 2022).

Specifically, the allele range for primer C11CANN1 observed in this study (155–164 bp) closely corresponds to the ranges reported (138–180 bp). Both B05CANN1 (242–245 bp) and B01CANN1 (323–326 bp) were within expected ranges of 217–256 bp and 314–460 bp, respectively, as well as ANUCS303 (147–153 bp) overlapped with the literature (141–157 bp). Primer H09CANN2 (220 bp) fit within the known range (204–224 bp), and ANUCS304 (170–203 bp) and ANUCS201 (157–172 bp) were generally consistent with previous studies (146–230 bp). In contrast, primer ANUCS501 (220 bp) was notably outside the reported range (72–105 bp).

### Cannabinoids profile

Cannabinoids in the flower and extracts were identified using HPLC-PDA, based on retention times and UV spectra. This analysis is essential for the pharmaceutical development of cannabinoids, as these profiles directly influence downstream processing and product quality. SOMAÍ Pharmaceuticals prioritizes the extraction of Δ^9^-THC by utilizing cannabis flowers with a high cannabinoid concentration, ensuring the production of extracts rich in Δ^9^-THC. Chromatograms for each processing stage, namely, dried flowers, raw extracts, and purified extracts, were overlaid to compare the cannabinoid profile of the three cultivars and can be found in the supplementary materials (Figure S2).

The results revealed overlapped cannabinoid profiles, with minor differences across the cultivars. Cultivar B shows a higher CBN peak compared to those of cultivar A and cultivar C, which could be attributed to the age of the batch (as confirmed by the laboratory), as CBN is a known degradation product of ∆^9^-THC (Jaidee et al. 2022). Additionally, an unknown peak at 11.54 min is present in cultivar A and cultivar C but is absent in cultivar B, constituting less than 1% of the chromatogram area, therefore indicating a minor variation with little impact on the overall cannabinoid profiles. The In Process Control (IPC) tests performed during the industrial extraction and purification process show that the production steps don’t promote product degradation, since the total THC and CBN concentration remain stable for 24 months according to stability protocols and assays already completed in a GMP environment (NTA/NCA 2025).

A detailed examination shows that the cannabinoid profiles of cultivar A and cultivar C flowers are highly similar, with the most significant differences arising during the extraction process from flower to extract. These changes result from the decarboxylation process during extraction, where acidic cannabinoids like THCA, CBGA, and CBCA are converted to their active forms, THC, CBG, and CBC, respectively. Despite this transformation, the relative abundance of cannabinoids remains consistent across all stages, preserving the overall profile from raw material to final purified extract. Moreover, the chromatograms of purified extracts closely resemble those of raw extracts, indicating that distillation primarily concentrates cannabinoids while removing non-cannabinoid constituents like impurities, without introducing significant changes to the profile.

**Fig. 2:**
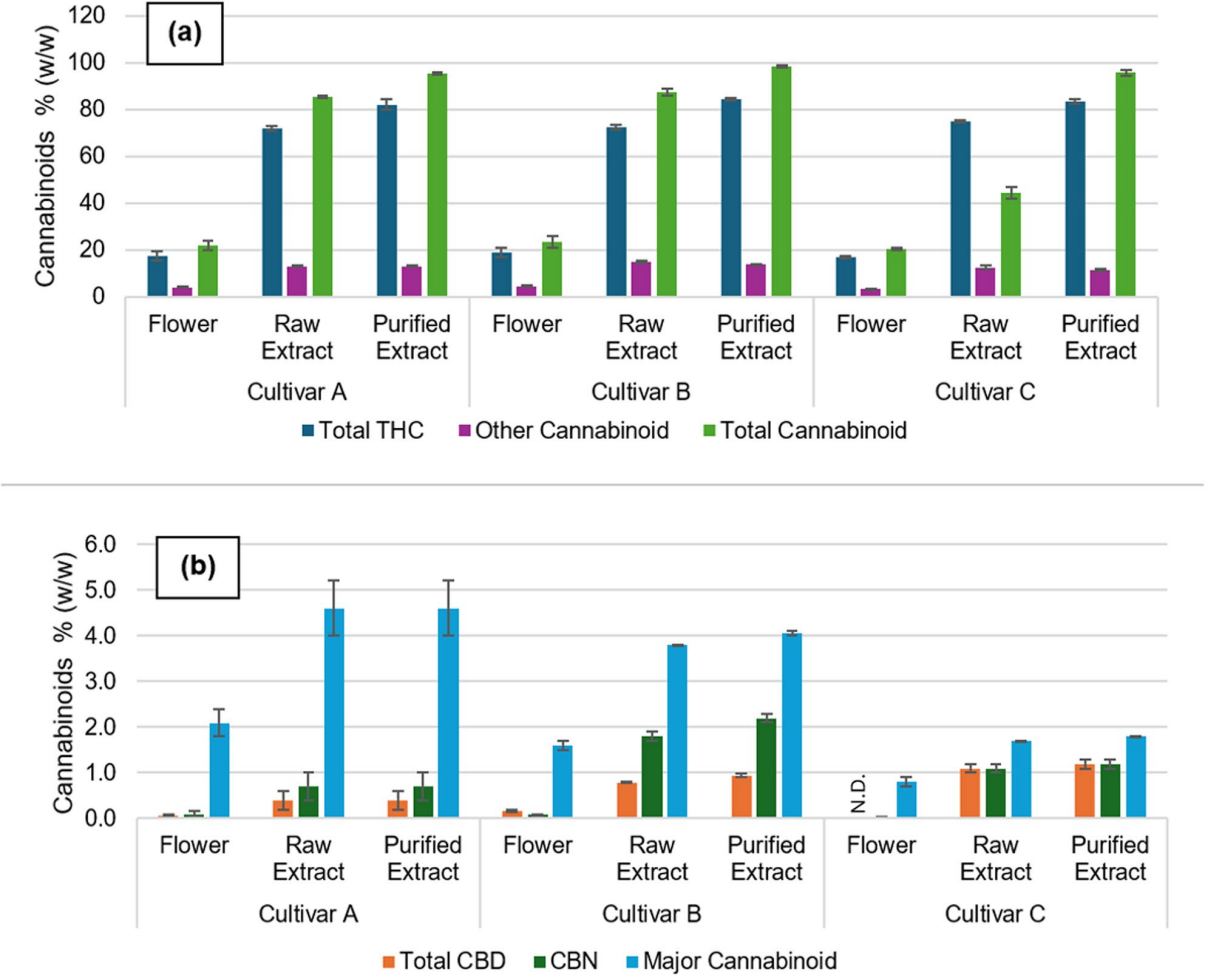
**a** Overview of total ∆^9^-THC, other cannabinoids, and total cannabinoids across different processing stages (flower, raw extract, and purified extract) for cultivars A, B, and C **b** Overview of total CBD, CBN, and major cannabinoids across the same processing stages for cultivars A, B, and C. Values are expressed as mean ± standard deviation of at least two batches. ND – not detected

After the distillation process, impurities such as phenolic compounds and solvent residues are eliminated based on their boiling points. In contrast, the chromatographic profile of the cannabinoids only shows variations in peak intensity, likely due to the similar boiling points of these compounds. These findings suggest that during the purification stage, the cannabinoids are carried over into the purified extract. To confirm this, the overall composition of cannabinoids in the products can be found in Fig. 2 and in more detail in Table S1 of the supplementary materials.

Figure 2 (a). reveals a significant increase in total ∆^9^-THC content from approximately 18% in the flower to 73% in the raw extract and 83% in the purified extract. A similar trend is observed for all cannabinoids presented in Fig. 2, with total cannabinoid content reaching 97% in the purified extract, reflecting the effectiveness of both extraction and purification in concentrating the desired compounds.

As with the chromatographic analysis, the cannabinoid profiles of flowers from different cultivars are consistent, with no significant deviation noted. The extraction and purification processes resulted in products with comparable cannabinoid profiles, reflecting the uniformity of the raw materials and the efficiency of the processing steps.

**Fig. 3:**
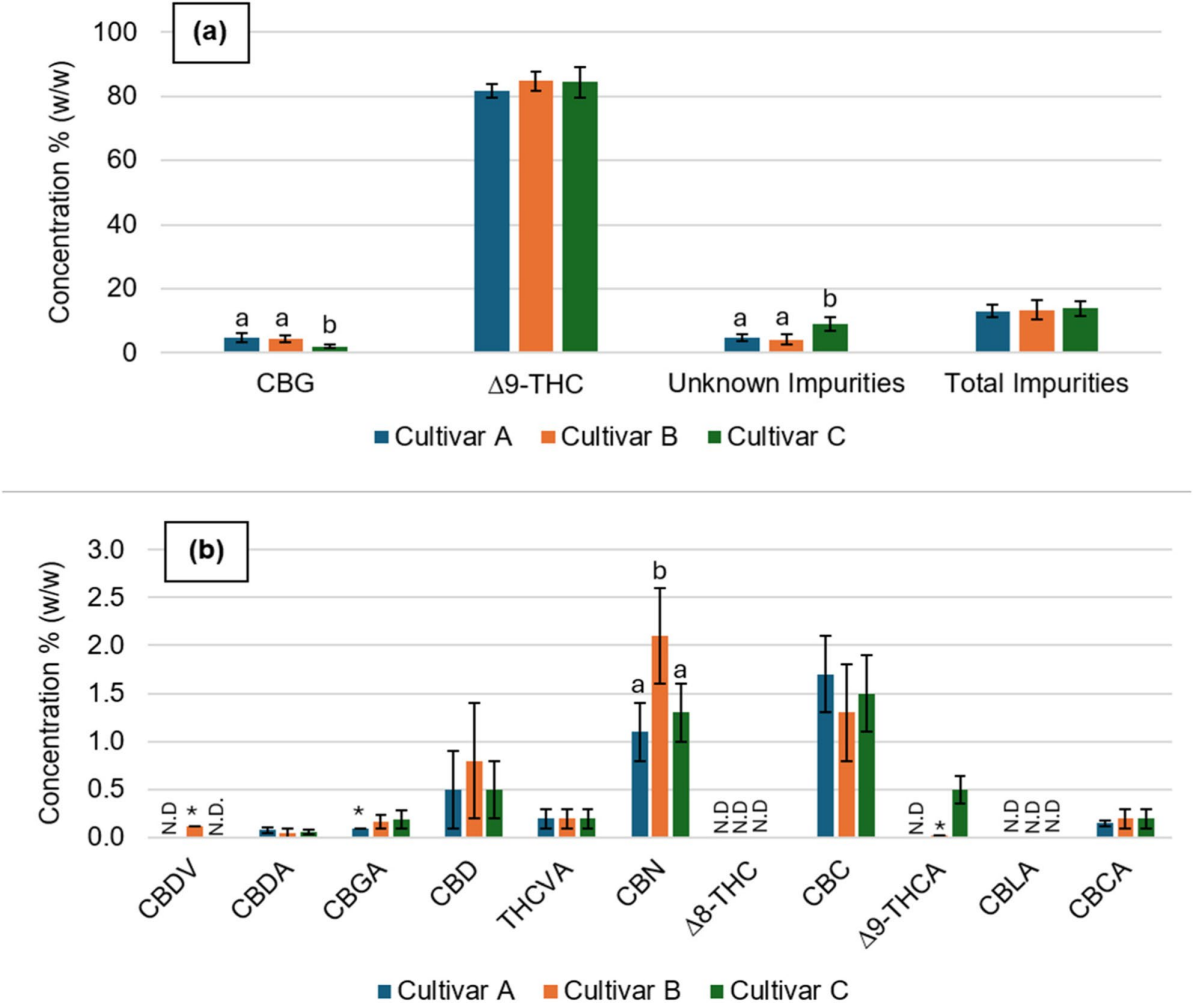
**a** Concentrations (% w/w) of Δ⁹-THC, CBG, unknown impurities, and total impurities in purified extracts from cultivars A, B, and C following up to five distillations **b** Concentrations (% w/w) of minor and precursor cannabinoids, including CBDV, CBDA, CBGA, CBD, THCVA, CBN, Δ⁸-THC, CBC, Δ⁹-THCA, CBLA, and CBCA, in the same samples

Further analysis was conducted to assess the cannabinoid composition of the distilled fractions obtained during the purification process. Figure 3 summarizes the distribution of individual cannabinoids across the different distillation fractions, providing insight into the separation and concentration processes during distillation. Detailed quantitative values and retention times are provided in Table S2 (Supplementary Materials).

Bars represent mean ± standard deviation (up to five distillations). Statistical differences between cultivars for each cannabinoid are indicated by different letters (a, b), with *p* < 0.05 considered significant (Kruskal–Wallis test followed by Dunn’s post hoc test, p-values adjusted using the Benjamini–Hochberg method). ND – not detected. *Values detected in only one of at least three replicates, so no error is reported.

Figure 3 presents the chemical profiles of cannabinoids for cultivars A, B, and C following up to five distillations, highlighting both major and minor cannabinoid content. All cultivars exhibited similar concentrations of major cannabinoids, with no significant differences observed between cultivars for the major cannabinoids such as Δ^9^-THC, CBDA, and CBD, indicating that genetic differences among these cultivars have minimal influence on the chemical composition following purification. Notably, CBG was identified as the second most abundant cannabinoid in the Δ^9^-THC-rich extracts.

Minimal differences were observed in cannabinoids like CBN, where cultivar B showed higher levels (2.1 ± 0.5%) compared to cultivar A (1.1 ± 0.3%) and C (1.3 ± 0.3%). This difference could be attributed to factors such as aging or storage conditions rather than inherent genetic variability, as CBN is a degradation product of THC.

Minor cannabinoids, which are typically more sensitive to genetic and environmental variations, highlight additional nuances. The cannabinoids CBDV, Δ^8^-THC, and CBLA are not detected in any of the cultivars, or are found in trace amounts, such as 0.12% of CBDV in cultivar B. This suggests that these cannabinoids are not prominent in these cultivars under the tested conditions, and as such, their presence is comparable across all three cultivars. CBGA concentrations are similar across all cultivars. Although cultivar A lacks a standard deviation due to only a single replicate being quantified, the values are of the same order of magnitude, indicating comparable quantities in all cultivars.

Cultivar C, however, demonstrates a distinct chemical profile, with lower CBG levels (2.0%) statistically significant (*p* < 0.05) compared to cultivars A and B (around 4.4% each) and higher levels of unknown impurities (8.9 ± 2.2%), suggesting unique environmental or genetic factors influencing its composition. Despite these differences, all cultivars exhibited a similar total non-cannabinoid content of approx. 13% of the plant, reinforcing the idea that the purification process is effective in concentrating cannabinoids while maintaining overall consistency.

Results highlight that, despite the differences in genotype among the cultivars, the chemotype remains similar among them. Cultivars A and B exhibit nearly identical cannabinoid profiles, suggesting that the genetic variations between their source materials do not change the chemical composition post-distillation. However, cultivar C stands out due to its lower CBG content, which is half that of the other cultivars, and its significantly higher levels of unknown compounds taken as impurities for a full cannabinoid extraction, approximately double those of cultivars A and B. These differences were statistically significant (*p* < 0.05).

These findings underscore that major cannabinoid profiles remain consistent across cultivars, particularly for A and B while genetic differences can influence minor cannabinoids and impurity levels.

This could be explained by differences in the expression or activity of enzymes like CBG synthase (Kim et al. 2022). Reduced activity or lower expression of CBG synthase in cultivar C may lead to the reduced production of cannabigerol (CBG), which could also result in a higher accumulation of unknown impurities, or if a plant expresses fewer enzymes such as tetrahydrocannabinolic acid synthase (THCAS) or cannabidiolic acid synthase (CBDAS), more cannabigerolic acid (CBGA) might be diverted into pathways for minor cannabinoids like CBC or CBG. In conclusion, the observed differences in minor cannabinoids across the cultivars can likely be attributed to differences in the activity or expression of enzymes involved in cannabinoid biosynthesis, such as THC oxidase for CBN, CBDV synthase for cannabidivarin (CBDV), and CBG synthase for CBG. These enzymatic factors, combined with potential genetic and environmental influences, may help explain the variations in cannabinoid concentrations and impurity levels across the cultivars (Sng et al. 2024).

While further investigation of these trace levels’ origins and implications is warranted, these findings reinforce the importance of combined genetic and chemical profiling to ensure product consistency and to better understand cultivar-specific characteristics.

### Non-Cannabinoid content

Non-cannabinoid compounds in *C. sativa*, such as phenolics, flavonoids, terpenes, waxes, and chlorophylls, play essential roles beyond the well-known cannabinoids like THC and CBD. Together, these compounds contribute to the plant’s pharmacological complexity, often described as the “entourage effect”, where multiple constituents are questioned to work synergistically for the enhancement of any therapeutic potential (André et al. 2024). For instance, phenolics are known to act as antioxidants, providing neuroprotective and anti-inflammatory benefits, and similarly, flavonoids possess antioxidant and anticancer properties (Cásedas et al. 2022). Waxes and chlorophylls, though important for plant protection and growth, are generally removed during extraction to improve the extract’s purity, taste, and appearance (Ciolino et al. 2018; Ribeiro et al. 2021). However, to date, no synergistic or complementary effect in cannabis appears to have been proven (André et al. 2024).

**Fig. 4:**
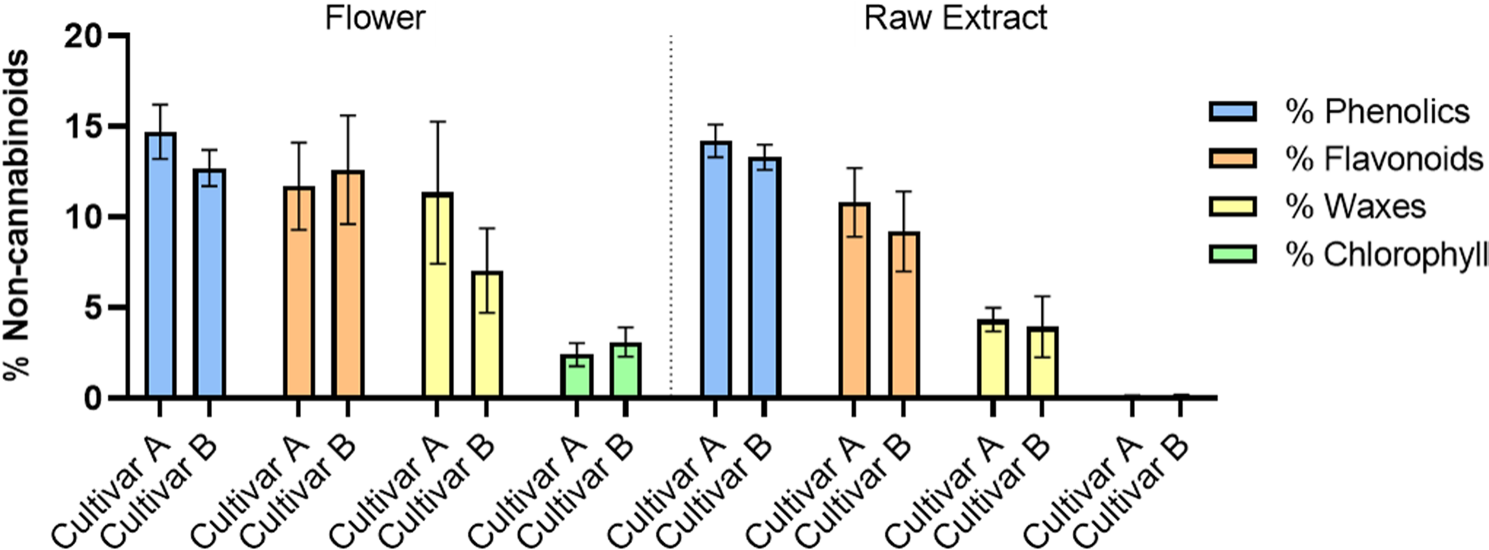
Percentage of non-cannabinoids in the flower and raw extract for cultivars A and B. Data were analyzed using the Kruskal-Wallis test followed by Dunn’s post-hoc test, with *p*-values adjusted using the Benjamini-Hochberg method. No statistically significant differences between cultivars for each group of non-cannabinoids were detected (*p* > 0.05)

Regardless of its effects, non-cannabinoid compounds can vary among the stages of cannabis processing, especially when going from flower to raw extract, as shown for cultivars A and B (Fig. 4). Hence, these compounds are key to comprehending possible chemical changes during extraction and their impact on the final product’s composition. The analysis highlights the retention and removal of these compounds throughout the process.

The results demonstrate the changes in the non-cannabinoid composition as the material progresses from the flower to the raw extract. Chlorophyll and waxes, which are abundant in the flower, show a significant reduction in the raw extract corresponding with the goals of processing, as these compounds are often considered impurities that can negatively affect the final product’s sensory properties. Phenolics and flavonoids are present at higher levels in the flower, with phenolics being particularly abundant. While cultivar A consistently exhibits slightly higher phenolic levels than cultivar B in both stages, the differences are not statistically significant, as also seen in flavonoids (*p* > 0.05). Although some overall reduction is observed during the extraction process, both compounds remain at detectable levels in the purified extract. The flavonoid/phenolic compounds ratio in cultivar A and cultivar B remains relatively similar throughout the process, maintaining an approximate 1:1 ratio, namely, in the flower, 0.8 for cultivar A and 1.0 for cultivar B, and in the raw extract (0.8 for cultivar A and 0.7 for cultivar B). This suggests that these compounds are relatively stable throughout the process, which is advantageous for retaining their antioxidant and anti-inflammatory properties in the final product.

However, accurate quantification of phenolics and flavonoids in complex matrices such as plant extracts might be challenging due to their overlapping chemical structures. Some flavonoids, particularly those lacking phenolic groups, can react with reagents commonly used for phenol detection (e.g., Folin-Ciocalteu), leading to overestimation of phenolics (Platzer et al. 2021). Conversely, the phenolic nature of THC can interfere with flavonoid measurements due to THC’s instability and oxidation to CBN in a redox environment, while CBD, which contains two phenolic groups, is less likely to be affected (Shraim et al. 2021). This overlap provides evidence of the inherent complexity of accurately quantifying these compounds using conventional methods. To address these limitations, precise quantification and identification of individual phenolic and flavonoid compounds may provide additional understanding of their relative contribution to the overall non-cannabinoid profile. However, preliminary data (unpublished results; Sassano 2024; NTA/NCA 2025) indicate that variations in the concentrations of more than one hundred non-cannabinoid metabolites, which may be present, are at trace levels, and it is difficult to provide a clear contribution. On the other hand, the possibility of variations in chemical profiles resulting from environmental factors rather than genetic differences should not be excluded, but controlling variables such as light exposure, temperature, and nutrient availability are ensured in cultivars for medical use. Namely, the herbal substance, Cannabis Flower (inflorescences), is obtained from qualified growers with GACP accreditation and previously audited for qualification purposes.

Within regulatory expectations, consistency of active pharmaceutical ingredients (APIs) across batches requires a clear link between product quality and clinical outcomes. Providing tools to control variability in natural-source products through genotype + chemotype stability enables to predict reproducible therapeutic effects, aligning directly with regulatory frameworks for botanical drugs.

This study explores the genotype (e.g., ANUCS303/ANUCS303/C11CANN1 PCR profiles) as a tool to identify and control cultivar sources and the chemotype (cannabinoid ratios) as a surrogate marker for pharmacological activity. The combined “identity fingerprint” is used to ensure therapeutic equivalence across batches. The preliminary results support a vision that efficacy is not only linked to dosage but also to source plant identity. Finally, this study is in line with (Pereira Da Silva Oliveira et al., 2024), on the importance of genetic variability in the medicinal use of cannabis. Genetic diversity among different cannabis cultivars is expected to result in distinct chemical profiles, influencing therapeutic efficacy and effects on patients. Furthermore, individual patient genetic factors may affect response to cannabis compounds, highlighting the need for a personalized approach in cannabis medicine. Lessons learned from Gosh et al. (2023) have already highlighted how monoecious plants’ self-pollination is associated with minimal variation in cannabinoid content from generation to generation. Understanding and controlling this variability is crucial in future studies to optimize therapeutic benefits and minimize potential adverse effects.

## Conclusion

The remarkable genetic and chemical diversity of *C. sativa* L. hybridized cultivars highlights their vast potential for the pharmaceutical industry, offering unique profiles that can be leveraged to develop consistent and targeted therapeutic applications. This study emphasizes the critical importance of integrating genetic and chemical profiling to achieve standardization and ensure robust quality control of *Cannabis sativa* cultivars. Notably, the findings underscore the potential of ANUCS303, ANUCS304 and C11CANN1 primers as valuable tools for cultivar genetic fingerprinting, contributing to the reliable supply of high-quality raw materials for medicinal cannabis production. These findings, while encouraging, highlight the need for further validation with a more diverse set of cultivars and a comprehensive set of primers to enhance the effectiveness and reliability of SSR-based cultivar identification. A consistency was found in major cannabinoids obtained across different batches of the same cultivar and among cultivars; conversely, only minor cannabinoids and impurities’ content demonstrated variability among different cultivars. Further investigation of these impurities is needed to better understand their implications. Non-cannabinoid compounds such as waxes and chlorophylls were efficiently removed during purification, ensuring different batches of the same cultivar maintain reproducibility of the binomial genetic/environmental conditions as reflected in the cannabinoids’ qualitative and quantitative profile of the chemotype. By demonstrating a potential critical interdependence between genotype identity and chemotype reproducibility, we provide a framework that meets regulatory expectations for botanical drug standardization and ensures consistent therapeutic efficacy in *Cannabis sativa*-based medicines.

Overall, this research provides a foundation for advancing the standardization of medicinal cannabis, offering insights into the genetic and chemical variability of *C. sativa* cultivars. Ultimately, these knowledge efforts should be expanded to more *C. sativa* populations to enhance the safety, efficacy, and reliability of cannabis-based therapeutics.

## Supplementary Information

Supplementary material 1.

## Data Availability

All data generated or analyzed in this study are available within this article and its supplementary files.

## Abbreviations

RAPD: Random amplified polymorphic DNA
AFLP: Amplified fragment length polymorphism
SSR: Simple sequence repeats
SNP: Single nucleotide polymorphisms
HPLC: High-performance liquid chromatography
PDA: Photodiode array detector
PCR: Polymerase chain reaction
CBDV: Cannabidivarin
CBDA: Cannabidiol acid
CBGA: Cannabigerolic acid
CBG: Cannabigerol
THCVA: Tetrahydrocannabivarin acid
CBC: Cannabichromene
CBLA: Cannabicyclolic acid
CBCA: Cannabichromenic acid
CBN: Cannabinol
THC: Tetrahydrocannabinol
THCA: Tetrahydrocannabinolic acid
Δ^8^-THC: (−)-Δ8-trans-tetrahydrocannabinol
CBD: Cannabidiol
