# Supplementary material for: Genotype and chemotype insights of high-THC medicinal Cannabis sativa L.: the role of SSR markers in the identification of cultivars
Source: J Cannabis Res. 2025 Nov 22;8:2. doi: 10.1186/s42238-025-00357-w (PMC12763954; doi:10.1186/s42238-025-00357-w)
Supplement: Supplementary file 1 — Supplementary material 1. [file 42238_2025_357_MOESM1_ESM.docx]

**Supplementary Materials**

**Genotype and chemotype insights of High-THC medicinal *Cannabis sativa* L.: the role of SSR markers in the identification of cultivars**

Ana Patrícia Gomes^1,2^(0009-0006-2286-9817), Sara Vicente^3^(0000-0002-8538-3586), Joana Rosa^2^, Iva Vinhas^2^, António Marques da Costa^2^, Michael Sassano^2^, Luis Monteiro Rodrigues^1^(0000-0003-0198-7671), Patrícia Rijo^1^(0000-0001-7992-8343), Helena Trindade^4,*^(0000-0002-1209-2622), Maria do Céu Costa^1^(0000-0002-9887-0973)

^1^CBIOS - Universidade Lusófona’s Research Center for Biosciences & Health Technologies, Campo Grande 376, 1749-024 Lisboa, Portugal

^2^SOMAÍ Pharmaceuticals, R. 13 de Maio 52, 2580-507, Carregado, Portugal

^3^NICiTeS, IPLUSO, ERISA-Escola Superior de Saúde Ribeiro Sanches, Lisboa, Portugal

^4^cE3c - Centre for Ecology, Evolution and Environmental Changes & CHANGE - Global Change and Sustainability Institute, Faculdade de Ciências, Universidade de Lisboa, Campo Grande, 1749-016 Lisboa, Portugal

*corresponding author email: mhdonato@ciencias.ulisboa.pt

Figure S1. Allele amplification size obtained following the analysis with the optimized SSR primers. In orange is cultivar A, in green is cultivar B and in blue is cultivar C. Each primer set was marked with a specific fluorescent dye for the capillary gel electrophoresis (Hex and 6-FAM).

Figure S2. Overlay of the chromatograms of the (I) flowers, (II) raw extract and (III) Δ^9^-THC purified extracts produced from different flower cultivars. In orange is cultivar A, in green is cultivar B and in blue is cultivar C.


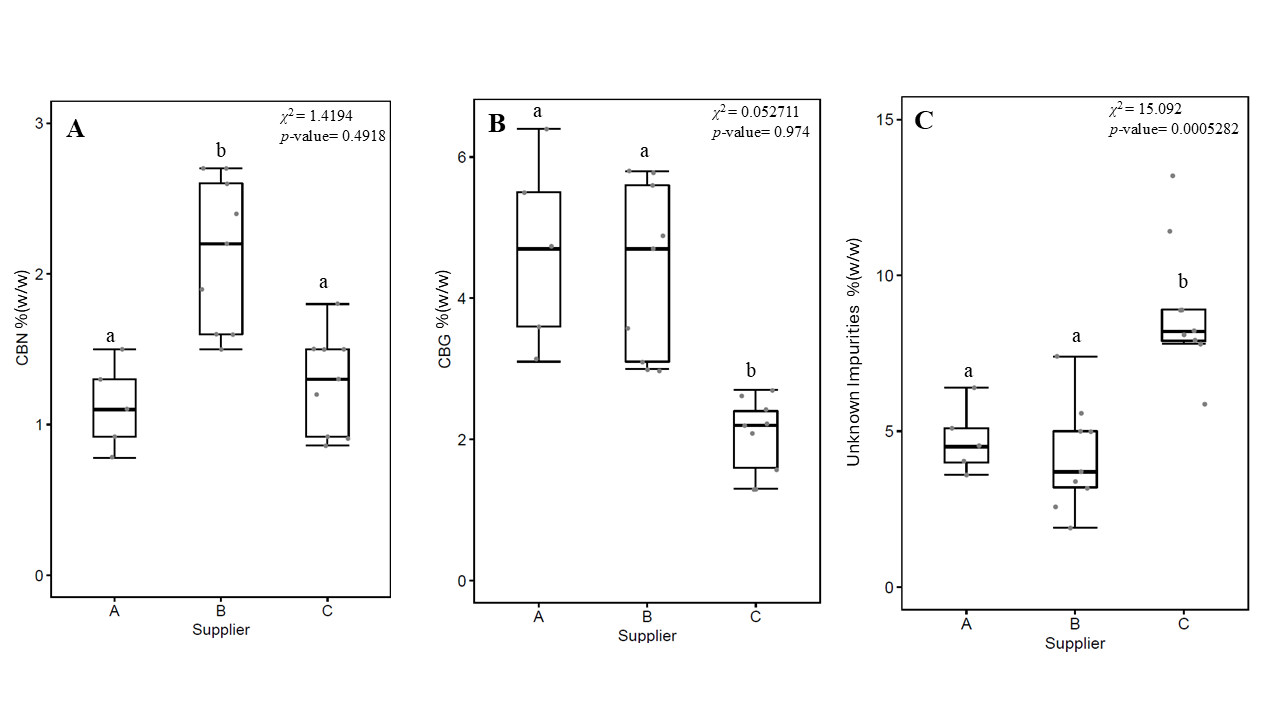
Figure S3. Box-plots showing (A) CBN, (B) CBG, (C) unknown impurities content across all suppliers in the purified extract. Different letters indicate significant differences (p<0.05) among groups based on Kruskal-Wallis test followed by Dunn post-hoc test in R statistical environment (R Core Team 2016).

Table S1. Overview of cannabinoids (% w/w) across different processing stages (flower, raw extract, and purified extract) for cultivars A, B, and C, determined as the mean ± standard deviation of at least 2 batches. ND - not detected.

| **Cultivar** | **Cannabinoids**  **% (w/w)** | **Total THC** | **Total CBD** | **CBN** | **Major**  **Cannabinoid** | **Other**  **Cannabinoid** | **Total**  **Cannabinoid** |
| --- | --- | --- | --- | --- | --- | --- | --- |
| A | Flower | 17.5±2.2 | 0.07±0.03 | 0.10±0.06 | 2.1±0.3 | 4.2±0.3 | 22.0±2.2 |
|  | Raw Extract | 72.1±1.1 | 0.4±0.2 | 0.7±0.3 | 4.6±0.6 | 13.2±0.4 | 85.3±0.5 |
|  | Purified Extract | 82.2±2.4 | 0.4±0.2 | 0.7±0.3 | 4.6±0.6 | 13.2±0.4 | 95.3±0.5 |
| B | Flower | 19.0±2.2 | 0.16±0.02 | 0.1±0.0 | 1.6±0.1 | 4.7±0.1 | 23.5±2.5 |
|  | Raw Extract | 72.4±0.9 | 0.79±0.02 | 1.8±0.1 | 3.8±0.0 | 15.1±0.5 | 87.5±1.5 |
|  | Purified Extract | 84.6±0.5 | 0.94±0.04 | 2.2±0.1 | 4.05±0.05 | 14.1±0.1 | 98.5±0.5 |
| C | Flower | 17.1±0.6 | ND | 0.033±0.005 | 0.8±0.1 | 3.5±0.1 | 20.3±0.5 |
|  | Raw Extract | 74.9±0.5 | 1.1±0.1 | 1.1±0.1 | 1.7±0.0 | 12.6±0.8 | 44.3±2.5 |
|  | Purified Extract | 83.7±1.0 | 1.2±0.1 | 1.2±0.1 | 1.8±0.0 | 11.7±0.5 | 95.7±1.2 |

Table S2. Quantification of individual cannabinoids (% w/w) in the purified extract from cultivars A, B and C, calculated as the mean ± standard deviation from up to five distillations. Data were analyzed using Kruskal-Wallis and Dunn’s post-hoc test with p-values adjusted using the Benjamini-Hochberg method. ND - not detected. Statistical differences between cultivars for each cannabinoid are indicated by different letters (a, b), with p < 0.05 considered statistically significant.

| **Peak** | **Retention time (min)** | **Cultivar A** | **Cultivar B** | **Cultivar C** |
| --- | --- | --- | --- | --- |
| CBDV | 2.73 | ND | 0.12* | ND |
| CBDA | 3.87 | 0.08±0.03 | 0.05±0.05 | 0.06±0.02 |
| CBGA | 4.11 | 0.1* | 0.17±0.07 | 0.19±0.10 |
| CBG | 4.40 | 4.7±1.4^a^ | 4.4±1.2^a^ | 2.0±0.5^b^ |
| CBD | 4.58 | 0.5±0.4 | 0.8±0.6 | 0.5±0.3 |
| THCVA | 6.00 | 0.2±0.1 | 0.2±0.1 | 0.2±0.1 |
| CBN | 6.55 | 1.1±0.3^a^ | 2.1±0.5^b^ | 1.3±0.3^a^ |
| ∆9-THC | 7.88 | 81.6±2.0 | 84.7±3.1 | 84.3±4.8 |
| ∆8-THC | 8.27 | ND | ND | ND |
| CBC | 9.70 | 1.7±0.4 | 1.3±0.5 | 1.5±0.4 |
| ∆9-THCA | 9.80 | ND | 0.02* | 0.5±0.14 |
| CBLA | 10.80 | ND | ND | ND |
| CBCA | 11.00 | 0.15±0.03 | 0.2±0.1 | 0.2±0.1 |
| Unknown Impurities | NA | 4.7±1.1^a^ | 4.2±1.7^a^ | 8.9±2.2^b^ |
| Total Impurities | NA | 13.0±1.9 | 13.4±3.1 | 13.8±2.4 |

*Values detected in only one of at least three replicates, so no error is reported.
